# An Overview of Published Articles in *Archives of Academic Emergency Medicine* in 2021

**DOI:** 10.22037/aaem.v10i1.1555

**Published:** 2022-02-27

**Authors:** Mehrnoosh Yazdanbakhsh, Somayeh Saghaei Dehkordi

**Affiliations:** 1 Journal Office, Emergency Ward, Shohadaye Tajrish Hospital, Shahid Beheshti University of Medical Sciences, Tehran, Iran.

***Archives of Academic Emergency Medicine*** has published 70 articles in 2021, which have been authored by more than three hundred researchers from various countries, including but not limited to the United States, Australia, India, Japan, Thailand, Iraq, Pakistan, France, Greece, and Iran. In this editorial, we intend to provide an overview of our publications in 2021, so that we can identify our strengths and weaknesses and provide a brief report on our performance to readers and authors, which they might find useful in becoming more familiar with the journal.

Just like the previous year, the published papers are indexed in SCOPUS, Web of Sciences, PubMed, and some other databases specified on the journal’s website. 

However, the Scopus CiteScore for the journal improved from 2.4 in 2020 to 4.5 in 2021 (https://www.scopus.com/sourceid/21101017909?origin=resultslist). The acceptance rate has been 18.81% in 2021 and reaching the decision to accept the articles has taken an average of 54 days. Out of the 70 published papers, 41 (58.57%) were original articles, 14 (20.0%) were review articles, 8 (11.42%) were case reports, and 7 (10.0%) were letters to editor. 

All published articles were matching the emergency medicine scope and were in line with journal commitments. 

Since the COVID-19 pandemic was still an issue throughout 2021 and there were still unknown aspects about this disease, researchers wanted to report new findings of the disease and its patterns and consequences, as well as its management. Thus, by publishing studies in this field, we tried our best to make these insights available to all health care providers around the world. So that they could learn from the experiences of each other and become more equipped to face the disease efficiently and make the lives of those affected by the disease in any way easier during these difficult times. Twenty-one (30.0%) of the published articles were related to COVID-19. Eleven articles were original research papers (1-11), 3 articles were letters (12-14), 2 were case reports (15, 16), and Five articles were review studies (17-20).

The 49 remaining articles, which consisted of 30 original articles, 9 review articles, 6 case reports, and 4 letters were on different topics. The letters consisted of 2 reports of preliminary studies performed (21, 22) and 2 expert views on different matters (23, 24). The letters are always reviewed by the editor and they are reviewed by external reviewers in case of presenting the findings of a study.

Among the case reports, 1 was on possible complications arising after treatments (25) and 5 reported unusual presentations of diseases (26-30). These reports were published to raise other physicians’ awareness of those situations so that they can either prevent facing the same situation or be able to better manage the situation if they encounter it.

Only one of the review articles was a narrative one (31) and the rest were systematic reviews and/or meta-analyses. Systematic reviews and meta-analyses are ranked as the highest level of evidence among articles and may be able to provide an answer to a burning question (32-38). Therefore, it is not surprising to see that 7.14% of this year’s review articles were related to COVID-19.

In this journal, we aim to introduce novel electronic approaches helping the management of patients. Machine learning is one of the novel fields in medicine, which may transform the practice of medicine, in 2021 we published 2 articles in that field (39, 40). 

Our aim in this journal is to play a part, even if it is a small one, in helping physicians do their practice of medicine, which can consequently improve the general health of all societies. This will not be possible without the hard work of researchers who perform the studies, so we would like to thank all the authors who trusted us with their valuable works throughout 2021. Our goal is to keep improving and continue to publish quality articles by authors from all over the world and everybody is invited to join us. 
